# Critical elements in nursing graduates’ transition to advanced practice roles and their perceived impact on patient care: an exploratory, descriptive study of graduates’ and their managers’ perceptions

**DOI:** 10.1186/s12912-022-00907-0

**Published:** 2022-05-20

**Authors:** Janita P. C. Chau, Suzanne H. S. Lo, Simon K. Y. Lam, Ravneet Saran, David R. Thompson

**Affiliations:** 1 The Nethersole School of Nursing, Faculty of Medicine, The Chinese University of Hong Kong, Shatin, New Territories, Hong Kong, China; 2 School of Nursing and Midwifery, Queen’s University Belfast, Belfast, UK

**Keywords:** Advanced practice nursing, Qualitative research, Quality of care

## Abstract

**Background:**

While there is a growth in the number of advanced practice nurses, there is a dearth of research examining their role transition from registered nurses. This study aimed to identify critical elements in the career path of nursing graduates who have taken up advanced practice roles and examine their perceived impact on patient care.

**Methods:**

An exploratory descriptive study was performed. Individual semi-structured interviews were conducted face-to-face with 10 nursing graduates now in advanced practice roles, and their ten respective managers. All interviews were audio-recorded and transcribed verbatim for latent and manifest content analyses.

**Results:**

The nursing graduates, six of whom were male, had a mean age of 35 years. All possessed a master’s degree and formal post-registration education and/or training. Six had at least three years’ experience as an advanced practice nurse. The managers, all female, had a mean age of 49 years. Eight had at least one year’s experience in their current position. Six key themes emerged: prior enhancement of personal and professional knowledge and skills; active participation in clinical roles and knowledge translation in preparation for advanced practice; adapting to new and diverse advanced practice nursing responsibilities; role of advanced practice nurses in leadership; personal qualities crucial for success in advanced practice; and provision of evidence-based patient-centered care.

**Conclusions:**

Postgraduate education, management knowledge and leadership skills, and active participation in clinical roles and knowledge translation appear crucial ingredients for promotion of nursing graduates to advanced practice roles. Other ingredients include a positive outlook, flexibility and adaptability, and good interpersonal, communication and problem-solving skills.

## Background

Ageing populations, chronic illnesses, and emerging infectious diseases are major health problems burdening healthcare systems globally [1]. A dynamic, versatile, and highly skilled nursing workforce is crucial to meet these rising healthcare demands [2]. University nursing education plays a key role in cultivating and maintaining a high-quality nursing workforce. With an emphasis on independent critical thinking, effective communication, and core clinical and problem-solving skills, university nursing graduates are expected to deliver holistic, individualized, and patient-centered care [3–5].

To keep pace with rapid change, roles have expanded beyond the provision of bedside nursing care [6]. According to the International Council of Nurses (ICN), advanced practice nurses (APNs) are registered nurses (RNs) who commonly hold a master’s degree and possess expert knowledge, complex decision-making skills, and clinical competencies for nursing practice that expands beyond the basic scope [7]. Apart from offering a high standard of care in practice, advanced practice roles include providing expert coaching, engaging in research, and demonstrating professional leadership [8]. In Hong Kong, APN roles were first piloted in 2003 and do not require a master’s degree [9]. Specific roles and responsibilities of APNs in Hong Kong encompass leadership and supervision, the development of professional nursing standards and protocols, the provision of complex and specialized patient care, clinical expertise, training, the initiation of and participation in evidence-based practice and nursing research, and the planning, implementation, and evaluation of new services. An expansion of nurse-led clinics by APNs has also been observed in recent years, with nurses managing up to 90% of patients for outpatient disease-specific care [9].

Evidence has shown the association of advanced practice with enhanced quality of care, patient satisfaction, and reduced hospital readmissions and mortality [10, 11]. However, the journey to gain competency and assume advanced practice roles can be challenging. According to the model of skill acquisition, it takes baccalaureate nursing graduates at least five years to pass from advanced beginner level of proficiency to expert in nursing practice [12]. Nursing students have also been recommended to begin strategically thinking about the direction of their careers from the undergraduate stage and seek appropriate collegial support and mentoring [13]. A scoping review of 54 studies found that facilitators and barriers to the implementation of advanced practice roles in primary care settings included colleagues’ understanding and acceptance of the role, nurses’ self-confidence, interdisciplinary collaborative relationships, organizational support, and leadership [14]. Studies also found that the transition journey could be distressing as nurses experienced feelings of inadequacy and role ambiguity due to new job expectations [15, 16].

A qualitative study of 18 nurses identified various skills that promote their career advancement, including interpersonal skills, professional competencies, and work commitment [17]. Yet, promising interventions to facilitate this transition can only be developed after gaining a context-specific understanding of factors influencing the role transition from RN to APN. Current data are mainly related to factors influencing role development or experiences of novice practitioners in establishing advanced practice roles. A significant knowledge gap exists, as there is a dearth of studies specifically looking at the career progression of nursing graduates towards advanced practice roles in Hong Kong. An exploration of the preparation for and challenges amid role transition of nursing graduates in advanced practice roles, and perceived impact of role transition on patient care is therefore warranted. This would help provide insight for the enhancement of the baccalaureate nursing curriculum to better prepare nursing students for future advanced practice roles, as well as allow for the improved integration of opportunities in early nursing careers to facilitate the development of APN competencies.

## Methods

### Aims

This study aimed to identify critical elements in the career path of nursing graduates who have taken up advanced practice roles and examine, from both graduates’ and their managers’ perspectives, their impact on patient care.

### Study design and participants

An exploratory descriptive research study was conducted with 10 nursing graduates now in advanced practice roles, and their 10 respective managers.

### Data collection

Individual in-depth semi-structured interviews were conducted face-to-face with a purposive sample of ten nursing graduates who had taken up advanced practice roles in four public hospitals, and their ten respective managers. Two interview guides based on the literature were developed. The first two authors, highly experienced in conducting qualitative interviews, interviewed the graduates and managers, respectively. All interviews were conducted in Cantonese in a private area at the participant’s convenience and lasted one hour on average. The interviews were audio-recorded and duplicated to avoid accidental data loss. Data saturation was achieved after interviews with around eight nurses and nine managers. Demographic data of participants, including age, gender, highest educational level, current position, years of experience as an RN, an APN, or in current position, and post-registration education, were also recorded.

### Data analysis

Both latent and manifest content analyses were conducted on the transcribed interviews. In latent content analysis, the transcripts were reviewed in a “segment-by-segment” manner to identify any underlying meanings or themes, and commonalities and differences, coded, and grouped into categories and subcategories via inductive reasoning. In manifest content analysis, data were quantified by counting the frequency of particular themes and ideas based on the actual words used by the participants for simple descriptive statistics.

Content analysis was conducted in four steps, namely decontextualization, recontextualization, categorization, and compilation [18]. Decontextualization involved familiarizing oneself with the data by repeatedly reading through the transcripts and inductively coding the text repeatedly to enhance the stability and reliability of the codes. In recontextualization, one researcher (RS) went over the transcribed text again to review the codes assigned in relation to the aim, as well as consider whether there was a need to code the unmarked text. In categorization, the codes were condensed and divided into domains based on the research question. Latent meaning was extracted, and themes and sub-themes were established. Identified themes and sub-themes were internally homogeneous and externally heterogeneous. Finally, in compilation, illustrative quotes were selected for each theme, and quantitative content analysis was conducted to quantify the themes. To increase validity of the findings, the first two co-authors (JPCC and SHSL) went over the transcripts and results to judge whether they were reasonable.

### Ethical considerations

Ethical approval was obtained from the Survey and Behavioral Research Ethics Committee of The Chinese University of Hong Kong (SBRE-086–12). The study adhered to local laws, the Hong Kong Personal Data (Privacy) Ordinance, the Declaration of Helsinki, institutional policies, and the ICH-GCP. Prior to data collection, participants were explained the study, and their right to confidentiality and to refuse participation at any time. Written informed consent was obtained. All data were kept strictly confidential and used for research purposes only.

## Results

The nursing graduates, six of whom were male, had a mean age of 35 years. All had a master’s degree and formal post-registration education and/or training. Six had at least three years’ experience as an APN (Table 1). The managers, all female, had a mean age of 49 years. Eight had at least one year’s experience in their current position (Table 2). Six key themes, similar among graduates and managers, emerged from the interviews.

**Table 1:** Demographic data of ten nursing graduates who have taken up advanced practice roles

Characteristic	No. (%)
Age (years)	
Mean (SD)	34.80 (1.55)
Range	31–36
Gender	
Male	6 (60.0)
Female	4 (40.0)
Highest educational level	
Master’s degree	10 (100)
Years of experience as an RN	
Mean (SD)	9.53 (1.34)
Range	8–12
Years of experience as an APN	
< 1	3 (30.0)
1–2	1 (10.0)
3–4	6 (60.0)
Post-registration education and training	
Postgraduate diploma	3 (30.0)
Post-registration certificate course	8 (80.0)
Overseas training	3 (30.0)

*Note. APN* = Advanced practice nurse; *RN* = Registered nurse; *SD* = Standard deviation

**Table 2:** Demographic data of the nursing graduates’ ten respective managers

Characteristic	No. (%)
Age (years)	
Mean (SD)	49.43 (6.29)
Not given	3 (30.0)
Range	41–59
Gender	
Female	10 (100)
Highest educational level	
Bachelor’s degree	1 (10.0)
Master’s degree	9 (90.0)
Current position	
APN	1 (10.0)
WM	8 (80.0)
DOM	1 (10.0)
Years of experience in current position	
< 1	2 (20.0)
1–5	4 (40.0)
6–10	2 (20.0)
> 10	2 (20.0)

*Note. APN* = Advanced practice nurse; *DOM* = Departmental operations manager; *WM* = Ward manager

### Theme 1: Prior enhancement of personal and professional knowledge and skills

#### Higher education and continuing professional development

All graduates and managers stressed the importance of higher education for nurses’ post-registration before accepting advanced practice roles. A health-related master’s degree was favored. Specialty-specific qualifications and overseas training courses were also highlighted, including post-registration certificates/diplomas. Participants reported the benefits of such education in developing their research and writing skills, such as literature searches, quantitative and qualitative analyses, and report writing, all likely to strengthen their capacity for evidence-based practice and performance in APN roles, including developing nursing standards:

*“I did a master’s in health education. It had a research component…I did some qualitative research…realize it is quite useful when trying to make changes in the ward now…I can better evaluate things in both [my] teaching and clinical [roles].”* (Graduate 3).

#### Management knowledge and leadership skills

Half of the graduates and most (eight) of the managers felt that enhancing their knowledge and skills in ward and people management was vital for success in an advanced practice role. Participants emphasized the importance of early preparation of RNs for understanding issues such as hospital and ward systems, workforce planning and allocation, budgeting, healthcare informatics, handling patient complaints and teambuilding:

*“I had to manage the ward…including resources, staff, the budget…I really didn’t know how to do all of these and had to learn them on the go.”* (Graduate 8).

### Theme 2: Active participation in clinical roles and knowledge translation in preparation for advanced practice

All participants mentioned making an active contribution to the ward and hospital as an essential factor in the promotion of RNs. They suggested that RNs should avail themselves of opportunities to participate in patient education programs, continuous quality improvement (CQI) initiatives, evidence-based projects, staff and student mentoring programs and nurse-led clinics. However, some graduates emphasized the difficulty in initiating these themselves and the need for managerial support. Half of the managers also expressed a willingness to create opportunities for graduates, learn of their ambitions and support their career aspirations:

*“If the ward manager does not give you opportunities, you can’t do much…they usually look at performance to give opportunities…so depends on your own effort.”* (Graduate 7).

In addition, four of the participants highlighted that greater involvement in inter-hospital and interdisciplinary meetings and departmental social activities were important ways for nurses to establish connections and build positive reputations among staff:

*“If you want to be promoted, you need to let others know who you are.”* (Manager 4).

### Theme 3: Adapting to new and diverse advanced practice nursing responsibilities

#### Changes in scope of practice

On promotion to advanced practice roles, both graduates and managers indicated a sharp increase in the scope of their work, with responsibilities ranging from clinical work supervision, inventory management, and bed coordination and assignment to teaching obligations, departmental website management, interdisciplinary collaboration, and clinical audits:

*“You have many roles: leadership, CQI projects…role model…monitor staff and daily practice, whether anyone failed to adhere to guidelines, whether we should update guidelines based on latest evidence…write appraisals for supporting staff, junior RNs.”* (Manager 5).

#### Challenges in coping with the role expansion

Due to their lack of initial experience, participants warned that newly promoted APNs often faced difficulties in managing their time and coping with heavy work commitments. However, some managers expressed sympathy for the pressures APNs faced and a willingness to give them space to learn and adapt:

*“I try to create a lot of opportunities for my APNs to use their problem-solving skills…if they don’t do that well, I take the responsibility…write an email to apologize… I like to give staff the space to fail…Giving support as ward manager is very important…but regardless, the APNs themselves have to be committed to do their work.”* (Manager 6).

### Theme 4: Role of advanced practice nurses in leadership

All participants mentioned the critical role of APNs in ensuring day-to-day operations of the ward, particularly in supervising quality and patient safety, directing and monitoring staff performance, and facilitating interdepartmental and interdisciplinary communication. A third of managers cited that APNs needed to be familiar with the healthcare system’s overall vision, assume responsibility, and assert the upholding of care standards and compassion:

*“[APN’s] position has changed to a supervision or management role…you need to set the standard and refuse to be lenient regarding best practice…[and] patient safety.”* (Manager 5).

### Theme 5: Personal qualities crucial for success in advanced practice

#### Interpersonal and communication skills

All participants stressed the central importance of good communication and collaboration with colleagues, regardless of discipline or status, and the need for APNs to respectfully navigate and balance diverse interests, develop synergy among subordinates, and de-escalate conflicts between colleagues, patients, and families. All managers stressed the value of high emotional intelligence, maturity, and patience:

*“You need to be patient when dealing with patients’ families…they may have some unreasonable complaints…but you need to always keep your smile…be polite.”* (Manager 4).

#### Problem-solving abilities

Participants conveyed that APNs should be able to flexibly handle different situations and crises and actively suggest innovative solutions to promote safer clinical environments. They also stressed the importance of APNs possessing critical and analytical thinking skills to independently ensure appropriate decision making:

*“As an APN, if I see that something is not right…not efficient…I can actively change it while as an RN, you tend to be more passive…just follow the guidelines.”* (Graduate 9).

#### Positive mindset

A third of participants emphasized the importance of having a positive outlook and the physical and emotional resilience to deal with difficulties in advanced practice roles. They stressed that APNs should see failures as opportunities for self-improvement:

*“You need to be willing to learn from your mistakes…I will support you if your attitude is alright even if you make errors.”* (Manager 7).

### Theme 6: Provision of evidence-based patient-centered care

Most participants felt that high performing APNs were champions for facilitating knowledge translation to improve clinical standards, and to optimize effectiveness and efficiency by ensuring that staff were supported in their role to enhance patient-centered care:

*“Our staff feels quite relieved and happy when [the APN] is on duty…helps everyone… staff don’t feel scared to ask him if they have questions.”* (Manager 6).

In addition, most (80%) graduates and managers asserted that there were positive effects on patient safety and care quality due to advanced practice roles. Initiatives supported by APNs, including nurse-led clinics and post-discharge support, also helped to reduce the number of patient readmissions:

*“We need to build a patient safety-centered culture…sometimes some colleagues may not understand why they have to do something due to their mindset…so you need to strictly tell them about the principle behind it instead of just telling them the task.”* (Graduate 8).

## Discussion

This study of nursing graduates and their managers aimed to identify key elements that might be considered by RNs during their transition to advanced practice roles and their potential impact on patient care. Six key themes were derived from the interview data, namely prior enhancement of personal and professional knowledge and skills; active participation in clinical roles and knowledge translation in preparation for advanced practice; adapting to new and diverse advanced practice nursing responsibilities; role of advanced practice nurses in leadership; personal qualities crucial for success in advanced practice; and provision of evidence-based patient-centered care.

Our findings highlight that a key ingredient in role transition to advanced practice roles is the possession of a master’s degree, preferably nursing or health-related, and specialty-related education and training. Such preparation is likely to provide not only a sound educational and professional foundation, but also bolster the confidence and leadership and management abilities of an APN. Other important attributes of an APN include having a positive outlook, good communication, interpersonal and problem-solving skills, and being flexible and adaptable. Importantly, the role of managers in providing support to new APNs was stressed, as was the positive impact of effective advanced practice role performance on patient care.

The importance of higher education and continuing professional development is supported by previous studies, including a cross-sectional survey of 3,255 RNs and nurse practitioners in New Zealand, which found a positive association between postgraduate education at any level and time spent in advanced practice activities [19]. A systematic review of 42 studies on advanced nursing roles in the UK, USA, and Australia also found diverse APN role domains, including advanced clinical practice, practice development, education, research, consultation, and administration [20], which is consistent with our findings. Moreover, while our study identified managerial support as significant for new APNs in the face of challenges, other studies have additionally suggested the benefits of a formal orientation on role transition [21], as well as greater collaborative opportunities, interprofessional education, and problem-based learning [22].

There is some evidence that advanced practice roles are associated with positive patient outcomes, including a systematic review of 20 randomized controlled trials and 49 observational studies, which found that APNs provided safe, effective, and quality nursing care [23]. A cross-sectional study of 120 Hong Kong-based APNs also identified improvements in patient care quality and safety [24]. However, overall, there remains a lack of empirical research on the effects of attaining advanced nursing qualifications on nurse-sensitive patient outcomes, such as cross-infection rates, as highlighted by a recent systematic review of 20 studies [25]. While our study has similarly alluded to the positive impact of advanced practice on patient outcomes, robust research examining this association is needed.

There is a paucity of studies examining the transition of RNs towards advanced practice roles, especially in the Hong Kong context, where to our knowledge, no other studies assessing this transition have been conducted. Our study involves interviews with both nursing graduates and their managers, thereby providing a more holistic exploration of this transition. Moreover, as the role of the APN in Hong Kong has been modeled based on ICN guidelines, our findings show that the educational preparation pre-requisites and nature of practice of these roles largely align with the ICN’s Guidelines on Advanced Nursing Practice [7]. However, it may be beneficial to more clearly define and clarify the requirements for promotion to APN roles and desired competencies specific to Hong Kong to better manage nursing graduates’ role expectations and their psychological preparation for improved adaptation to these roles.

Our study has two clear limitations. Firstly, as an exploratory descriptive study of ten graduates and ten managers recruited by purposive sampling, we cannot generalize our findings. Secondly, the study is restricted to one geographic area and its nursing and healthcare system, and it is acknowledged that requirements and prerequisites for role transition to advanced practice roles may differ among countries [24]. Nonetheless, our study findings do provide some pointers that may be universal.

## Conclusions

Postgraduate education, management knowledge and leadership skills, and active participation in clinical roles and knowledge translation appear crucial ingredients for promotion of nursing graduates to advanced practice roles. Other ingredients include a positive outlook, flexibility and adaptability, and good interpersonal, communication and problem-solving skills. Our study findings have the potential to inform nursing curricula and future interventions to facilitate the preparation and transition of nursing graduates towards advanced practice roles that promote high-quality, safe, and effective patient care, however, future studies are necessary to examine such associations.

## Data Availability

The datasets used and/or analysed during the current study are available from the corresponding author on reasonable request.

## Abbreviations

APN: Advanced Practice Nurse
CQI: Continuous Quality Improvement
ICN: International Council of Nurses
RN: Registered Nurses
